# Detecting and managing complications in cataract patients

**Published:** 2016

**Authors:** Nick Astbury, Lily A Nyamai

**Affiliations:** Clinical Senior Lecturer: International Centre for Eye Health, London School of Hygiene and Tropical Medicine, London, UK.; Tutorial Fellow: Department of Ophthalmology, University of Nairobi, Nairobi, Kenya. **lilynyamai@gmail.com**

**Figure:**
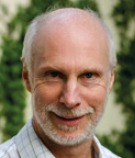
Nick Astbury

**Figure:**
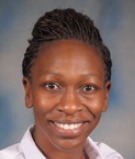
Lily A Nyamai

In order to ensure good cataract outcomes with the minimum of complications, the following are all essential:

- Well-trained staff
- Excellent teamwork
- Good pre-operative evaluation (including history taking, examination, investigations and biometry)
- Infection control (including prophylaxis)
- Functioning equipment
- Sufficient consumables (including intraocular lenses)
- Good postoperative care.

Even if these are all in place, problems can arise with a patient who can't keep still in theatre, an eye that is deep-set and difficult to access, a small pupil, weak lens zonules (whether due to pseudo-exfoliation or subluxation) or a hyper-mature cataract that requires a high degree of surgical skill. If the posterior capsule is ruptured and there is vitreous loss, there is a higher risk of postoperative complications such as endophthalmitis, retinal detachment or macular oedema. Poor vision postoperatively can be caused by uncorrected refractive error, particularly if no intraocular lens (IOL) was used or the wrong power IOL was inserted.

Things can also go wrong in the postoperative period if postoperative complications are missed, or if perioperative complications are not managed well. It is therefore important that all eye health workers who come into contact with the patient postoperatively know the basics of what the operation entails and what is normal so that they are alert to any signs or symptoms that might require action. They must know how to recognise an early or late complication and how to manage it effectively to prevent loss of sight – which we will cover in more detail in this article.

Complications are rare and in most cases can be treated effectively. In a small proportion of cases, further surgery may be needed. Very rarely, some complications can result in blindness.

**Figure:**
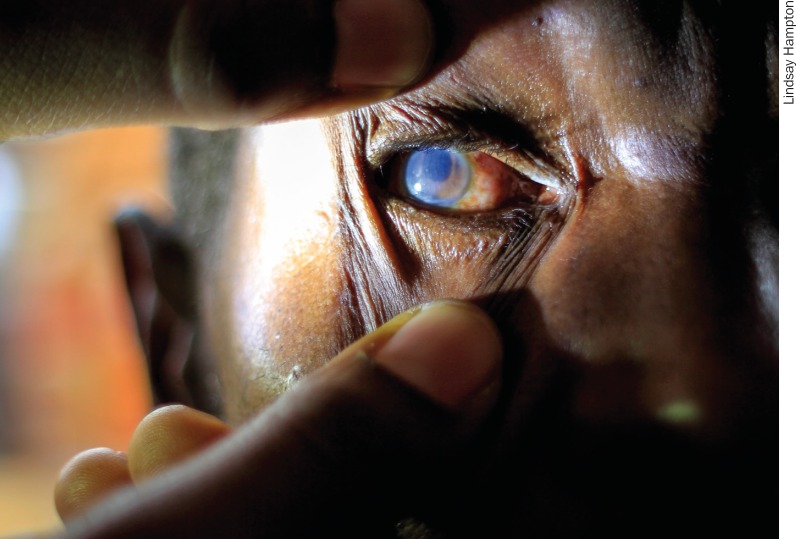
This patient has a hazy cornea and a peaked pupil following cataract surgery. There was also vitreous loss during surgery. KENYA

Some complications may arise despite a good initial surgical outcome but in most settings they can be avoided through effective communication between the eye team and the patients. Good rapport is needed with an honest discussion about expectations right from the start.

As a general rule, worsening sight, increasing pain, redness, swelling and discharge are all symptoms or signs that should trigger a referral.

What follows isa list of complications and advice on how to manage them in order to minimise the risk of a poor outcome.

## Early complications

These are complications which occur immediately following the operation (and may have their origin in the operation itself). With adequate vigilance and monitoring of patients postoperatively they can be detected and treated while the patient is still in the clinic. In addition, ensure that patients know they must alert a member of staff if:

- they experience pain (rather than slight discomfort)
- if their vision is reduced in any way
- if they notice any redness, swelling or discharge in their eyes.

**Discomfort.** Most patients will stay overnight before having their first dressing the next day. Some mild irritation can be expected which usually settles down over 1–2 days and the eyesight gradually improves. Severe pain is unusual and may indicate raised pressure in the eye or the start of an infection. If the eyesight is improving and the eye not unduly red and the discomfort is mild, simply reassure the patient that it will get better.

**Bruising or swelling of the eyelids/sub-conjunctival haemorrhage** may occur if a sub-Tenon's or peri-bulbar local anaesthetic injection has been given. It may take a week or ten days to settle. The patient can be reassured. Intraocular haemorrhage (hyphaema) caused by a bleeding wound or iris is rare. If significant or the intra-ocular pressure is raised, medical or surgical intervention may be required.

**Allergy to the steroid or antibiotic drops** prescribed postoperatively may rarely cause a reaction. Itching, local erythema and oedema around the eye may occur. Stopping the drops or using 1% hydrocortisone cream will allow it to settle.

**High pressure inside the eye.** A pressure spike postoperatively is common and may be due to retained visco-elastic. It usually settles without treatment. Patients with pre-existing glaucoma are more susceptible; therefore a review and pressure check on the day after surgery is advised. If you are in a surgical camp or you have reasons to suspect that patients may not return for follow-up, a short course of a beta blocker, such as Timolol, may be given.

**Low pressure inside the eye/leaking wound.** A larger or poorly constructed wound may sometimes leak, causing the eye to be soft. The eyesight may be blurred and there is an increased risk of infection. Referral and resuturing are likely to be required.

A **flat anterior chamber** postoperatively occurs mainly due to wound leak. Low intraocular pressure and a Sidel test will confirm a leak. Small leaks usually resolve spontaneously and can also be managed medically using cycloplegia, aqueous inhibitors and antibiotics, and by reducing steroid therapy. Alternatively, a tissue adhesive or a bandage contact lens may be applied. More significant wound leaks may need reformation of the anterior chamber and suturing.

However, sometimes a shallow anterior chamber occurs with high intraocular pressure. This is usually due to blockage of aqueous humour flow due to pupillary block. Pupillary block may be associated with postoperative uveitis resulting in synechiae of the iris to the vitreous, posterior capsule or IOL. This can result in a shallow anterior chamber and high intraocular pressure. Placement of an anterior chamber IOL without a prophylactic peripheral iridectomy can also result in pupillary block. A surgical or laser peripheral iridectomy, accompanied with frequent steroids, is usually effective.

**‘It is vital that patients monitor their own eye health and know where to go and what to do if they are concerned’**

**Clouding of the cornea** may occur after excessive surgical manipulation or if there is a pre-existing corneal dystrophy. Usually gradual clearing is expected over a few weeks or, rarely, months. In the rare cases that the cornea does not clear spontaneously, corneal transplant surgery may be necessary. Before referral for corneal oedema, topical steroids, hyperosmotic agents and a contact bandage lens may provide relief. However if the eye has little or no potential for vision then a Gundersen conjunctival flap may be performed.

**Figure 1.**
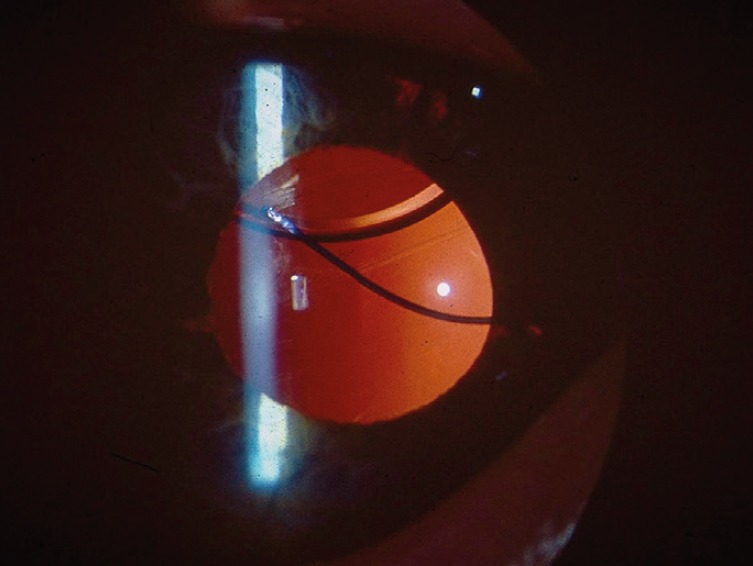
Decentred intraocular lens

**Decentration or dislocation of the implant (IOL) (Figure 1).** If the IOL haptics (loops) have been incorrectly placed, the IOL may be decentred. If the operation was complicated and the posterior capsule ruptured, the IOL may have fallen back into the eye. In either case, blurred vision or pain may be experienced and further surgery may be necessary.

In many cases, the IOLs used are rigid, one-piece acrylic lenses. A decentred or dislocated intraocular lens may either be intracapsular or extra-capsular. Intracapsular causes maybe due to capsular phimosis or inadequate zonular support, e.g. in pseudo-exfoliation. Extracapsular decentration occurs when either one or both haptics (loops) are located in the sulcus with the optic in the capsular bag. On some occasions the optic may be tilted or displaced in front of the iris in the anterior chamber. Management in these cases comprises observation for asymptomatic cases or dialling the intraocular lens centrally and ensuring stability. In cases where the zonules are inadequate an anterior chamber lens may have to be placed, or a single-piece lens sutured to the iris or sciera.

**Incorrect power of the implant.** Refractive ‘surprises’ (postoperative predicted errors greater than 2 diopters) occur in approximately 5% to 10% of lens implantations. Most are due to human error and are avoidable. Accurate preoperative biometry and strict adherence to protocol should prevent the wrong IOL being implanted. Refraction will reveal whether the IOL power has been miscalculated. Spectacle correction would normally allow the patient to benefit from the operation.

Postoperative refractive error is confirmed using retinoscopy and corrected using spectacles. This is usually done one month to six weeks postoperatively.

**Figure 2.**
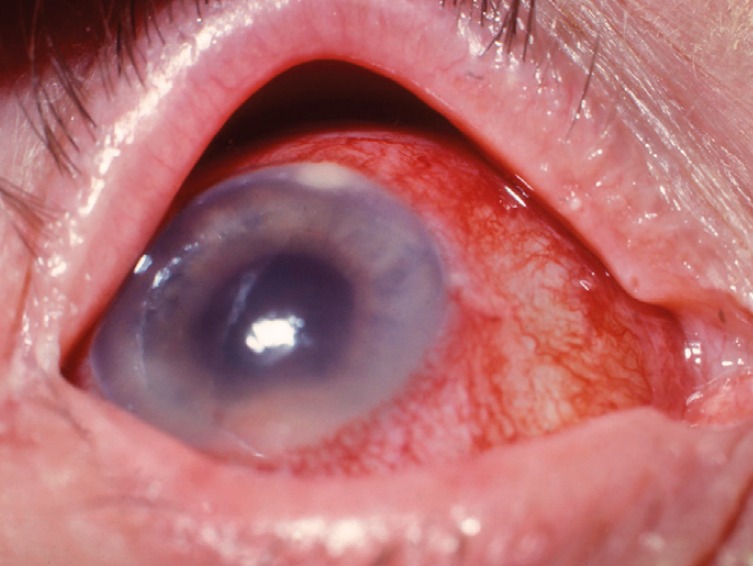
Endophthalmitis with hypopyon

**Infection in the eye (endophthalmitis) (Figure 2)** is the most serious complication with an incidence that varies from less than 1 in a thousand to several times that figure depending on the criteria of diagnosis, and whether the cases are culture-proven or clinically diagnosed.^1^ When acute, it develops in 2–5 days with pain being a prominent symptom. However, endophthalmitics can present up to 6 weeks after surgery. Ciliary injection (redness around the cornea) and conjunctival chemosis occur, and pus in the eye (hypopyon) may be visible in the anterior chamber. Immediate referral for culture and intravitreal antibiotics may save the eye. Read more online: **http://www.cehjournal.org/article/postoperative-endophthalmitis**

**Toxic anterior segment syndrome** (TASS) is a mimic of endophthalmitis. It is a severe sterile postoperative inflammation due to contaminated solutions used in surgery. Topical corticosteroids are given until the inflammation subsides. Frequent follow-up is also essential to monitor symptoms and reassess for bacterial infection and intraocular pressure.

## Late complications

These complications can occur after patients have gone home. It is therefore vital that patients monitor their own eye health and know where to go and what to do if they are concerned. A checklist of signs and symptoms can be sent home with patients.

**Figure 3.**
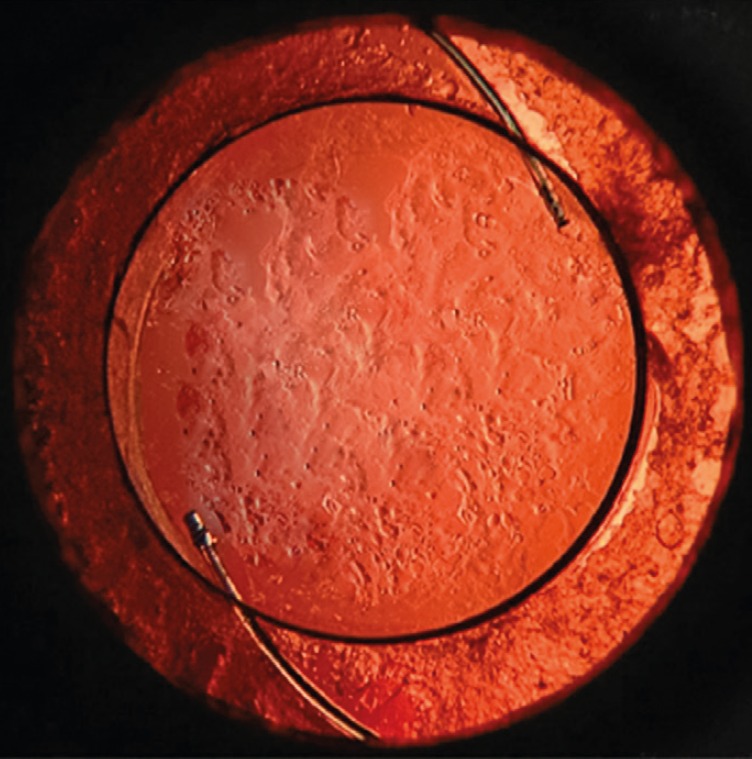
Posterior capsular opacification

**Cystoid macular oedema (CMO)** is often the cause of unexpected visual loss and may become evident 4–6 weeks after surgery. It is more likely if the operation has been complicated, or there is diabetic retinopathy or pre-existing macular scarring. The majority of cases resolve spontaneously over weeks or months but with some loss of contrast sensitivity or, more significantly, poor vision. CMO is often treated with topical/sub-Tenon's or intravitreal steroids or non-steroidal anti-inflammatory drops. Surgical intervention is called for when there is an identifiable provoking cause, for example a vitreous wick, retained lens fragments or a decentred intraocular lens. As a precaution, most patients suffering from diabetic retinopathy or epiretinal membranes (pre-existing scarring at the macula) should be given anti-inflammatory medication as prophylactic treatment after their operation. The symptoms are blurred or decreased central vision.

**Retinal detachment** may occur weeks or months after surgery, more commonly in highly myopic people or after complicated surgery with vitreous loss. The symptoms may include ‘flashes and floaters’ and a peripheral ‘shadow’ across the vision. Refer immediately.

**Posterior capsular opacification (PCO) (Figure 3)** occurs in 10% of patients after two years and is the commonest reason for further intervention after cataract surgery. It is caused by lens epithelial cells migrating across the (normally clear) posterior capsule of the lens. It is treated with Nd-YAG laser in the eye clinic. In young people and children, opacification can occur early and patients should be warned that this may occur. The symptoms are blurred vision and glare.

## Conclusion

The end of the operation is the beginning of an anxious period for the patient, when they are hoping that their sight will be restored. If complications have occurred the patient must be kept informed and the outlook must be explained to them. Postoperative symptoms should be heeded and signs carefully looked for in case intervention is required. Good preoperative counselling and awareness of postoperative problems will help to ensure that complications are detected early and managed effectively.
